# Ecological genomics of saprotrophy to biotrophy transitions in the genus *Clitopilus* (Fr. ex Rabenh.) P. Kumm. (*Agaricales*, *Entolomataceae*)

**DOI:** 10.3897/imafungus.17.179417

**Published:** 2026-01-27

**Authors:** Yuwei Zhang, Yuchen Wang, Irina S. Druzhinina, Fachada Vasco, Donglian Zhong, Long Peng, Jiajia Yao, Zhilin Yuan, Francis M. Martin

**Affiliations:** 1 State Key Laboratory of Tree Genetics and Breeding, Chinese Academy of Forestry, Beijing 100091, China Institute of Biotechnology, Zhejiang University Hangzhou China; 2 College of Forestry, Nanjing Forestry University, Nanjing 210037, China Royal Botanic Gardens Richmond United Kingdom; 3 Zhejiang Key Laboratory of Forest Genetics and Breeding, Research Institute of Subtropical Forestry, Chinese Academy of Forestry, Hangzhou 311400, China State Key Laboratory of Tree Genetics and Breeding, Chinese Academy of Forestry Beijing China; 4 Institute of Biotechnology, Zhejiang University, Hangzhou 310058, China Research Institute of Subtropical Forestry, Chinese Academy of Forestry Hangzhou China; 5 Royal Botanic Gardens, Kew, Richmond TW9 3AE, UK College of Forestry, Nanjing Forestry University Nanjing China; 6 Université de Lorraine, INRAE, UMR Interactions Arbres/Microorganismes, Centre INRAE Grand Est-Nancy, 54 280 Champenoux, France Université de Lorraine Champenoux France

**Keywords:** CAZymes, ecophysiology, IAA, nitrogen acquisition, pleuromutilin

## Abstract

Transitions between saprotrophic and biotrophic lifestyles represent pivotal evolutionary events in fungal ecology; however, the genomic and physiological mechanisms underlying such shifts remain poorly understood. The agaric genus *Clitopilus* (*Basidiomycota*, *Entolomataceae*) offers a valuable model system, with most species being soil saprotrophs. *Clitopilus cf. baronii* Consiglio & Setti exhibits genomic signatures suggesting incipient biotrophic capacity. Here, we investigated the genomic and eco-physiological properties of seven strains representing five *Clitopilus* species to identify traits associated with lifestyle transitions. ITS-based phylogeny combined with ecological metadata revealed potential facultative biotrophy in multiple taxa from the section *Scyphoides*. Physiological profiling showed that all strains utilized mannitol and sucrose poorly, preferred organic nitrogen compounds, and produced variable amounts of indole-3-acetic acid (IAA) *in vitro* in a strictly tryptophan-dependent manner. Enzymatic assays revealed substantial variations in the nitrogen and phosphorus acquisition capabilities among the strains. Comparative genomics of high-quality assemblies identified a pleuromutilin biosynthetic gene cluster (BGC) across all strains, although synteny analysis revealed considerable structural variation and putative gene loss, indicating that genomic plasticity potentially affects antibiotic production. Principal component analysis of carbohydrate-active enzymes (CAZymes) across 25 fungal genomes partitioned *Clitopilus* strains into two distinct groups: one resembling saprotrophic white-rot basidiomycetes, the other matching biotrophic ectomycorrhizal and endophytic taxa. This first comprehensive genomic analysis of *Clitopilus* revealed that nutritional specialization, phytohormone production, and CAZyme repertoire remodeling collectively signal an ongoing evolutionary transition from saprotrophy to plant-associated lifestyles in multiple lineages. These findings provide a rare genomic window into the early stages of symbiosis evolution, offering insights into how free-living fungi acquire the molecular toolkit for mutualistic partnerships.

## Introduction

*Clitopilus* (Fr. ex Rabenh.) P. Kumm. (*Basidiomycota*, *Agaricales*, *Entolomataceae*) is a genus of widespread saprotrophic fungi that forms small to medium basidiomata with convex to plano-convex caps, decurrent gills, and a distinctive pinkish spore print (Jian et al. 2020a; Peng et al. 2021, 2022). Recent taxonomic revisions based on morphology and molecular phylogeny have substantially clarified species boundaries within the genus (Noordeloos and Gates 2012; Jian et al. 2020a, 2025), with approximately 154 taxa currently recognized worldwide (Species Fungorum accessed 2025).

Understanding the evolutionary transitions between saprotrophic and biotrophic lifestyles represents a fundamental challenge in fungal ecology, with implications for ecosystem functioning, plant health, and the evolution of symbiosis. Such transitions are not uncommon in *Agaricales*, and several wood-decaying lineages, including *Mycena* species and other basidiomycetes, which have independently evolved facultative biotrophic capabilities, forming mutualistic associations with plants while retaining their saprotrophic potential (Smith et al. 2017; Harder et al. 2023, 2024). These lifestyle shifts are typically accompanied by genomic and physiological adaptations that enable successful plant colonization and nutrient exchange.

Recent evidence suggests that *Clitopilus* harbors transitional taxa. *Clitopilus cf. baronii* (formerly *C. hobsonii*) has been shown to establish mutualistic symbiosis with poplar trees *in vitro* under organic nitrogen conditions (Peng et al. 2022), suggesting incipient biotrophic capacity. This observation raises critical questions; is this lifestyle plasticity unique to *C. cf. baronii* or is it more widespread within the genus? Which genomic and physiological features underpin these transitions?

Genomic approaches offer powerful tools for addressing these questions. The composition of carbohydrate-active enzyme (CAZyme) repertoires, collectively termed the CAZome, has emerged as a promising indicator of fungal lifestyle and biotrophic potential (Lebreton et al. 2022; Looney et al. 2022; Martin and van der Heijden 2024). Shifts from saprotrophy toward biotrophy are typically characterized by: (1) contraction or loss of lignocellulose-degrading enzyme families (e.g., GH6, GH7, AA9) and certain auxiliary activities involved in plant cell wall breakdown; (2) retention or expansion of chitinases facilitating microbial interactions and fungal cell wall remodeling; and (3) metabolic reorientation toward storage carbohydrate utilization and nutrient exchange rather than plant biomass deconstruction (Lebreton et al. 2022; Looney et al. 2022). Beyond CAZymes, other genomic features, including secondary metabolite biosynthetic potential and nitrogen metabolism capabilities, may also reflect ecological strategies (Miyauchi et al. 2020).

One particularly intriguing trait in *Clitopilus* is the production of pleuromutilin, a diterpenoid antibiotic with potent antibacterial activity, which has been documented in several species (Hartley et al. 2009; Schafhauser et al. 2022). The biosynthetic capacity for specialized metabolites may be related to competitive interactions in soil environments or potentially to symbiotic signaling, although these connections remain unexplored.

Here, we integrated comparative genomics, physiological profiling, and enzymatic characterization across seven strains, representing five *Clitopilus* species, to test the hypothesis that ecological lifestyle transitions in *Clitopilus* are associated with coordinated genomic and physiological adaptations. Specifically, we (1) reconstructed phylogenetic relationships and assessed the ecological contexts of the studied taxa; (2) characterized nutritional preferences and enzymatic capabilities related to nitrogen and phosphorus acquisition; (3) quantified the production of IAA, a phytohormone relevant to plant-fungal interactions; (4) conducted comparative genomic analyses of CAZyme repertoires across saprotrophic and biotrophic basidiomycetes; and (5) characterized pleuromutilin biosynthetic gene clusters to assess antibiotic production potential. This integrative approach provides the first comprehensive genomic and physiological characterization of the *Clitopilus* clade, revealing evidence for multiple independent transitions toward plant-associated lifestyles and illuminating the mechanisms underlying fungal symbiosis evolution.

## Materials and methods

### Fungal strains used in this work

Information on the seven *Clitopilus* strains is presented in Table 1. Strains QYL-10 and NL-19 (*Clitopilus cf. baronii* Consiglio & Setti, formerly *C. hobsonii* (Berk.) P.D. Orton) and strain HSL (*Clitopilus cf. prunulus* (Scop.) P. Kumm.) were isolated from the ectomycorrhizal tips of *Quercus lyrata* Walter, *Q. michauxii* Nutt., and *Q. stewardii* Rehder, respectively. Strain XST (*Clitopilus cf. baronii* Consiglio & Setti) was isolated from the roots of *Cymbidium* Golden Sw. Strain CAB (*C. abprunulus* S.P. Jian, M. Karadelev & Zhu L. Yang), strain 84496 (*C. prunulus* (Scop.) P. Kumm.) and strain 50334 (*Clitopilus cf. scyphoides* (Fr.) Singer) were isolated from the fruiting body tissues. Of these, strain CAB was donated by Professor Yang Zhuliang (Kunming Institute of Botany, Chinese Academy of Sciences), and the latter two were obtained from a public culture collection. Other strains were deposited in the public culture collection. The fungi were routinely sub-cultured on potato dextrose agar (PDA; 200 g·L^-1^ potato, 20 g·L^-1^ glucose, 15 g·L^-1^ agar) at 25 °C in the dark.

**Table 1.** The basic information of *Clitopilus* strains used in this study.

Species	Strain names	Origin	Accession or collection numbers
*C. abprunulus*	CAB	Fruiting body	HKAS 107042
*Clitopilus cf. baronii*	QYL-10	ECM root tips of *Quercus lyrata*	CGMCC 20208
*Clitopilus cf. baronii*	NL-19	ECM root tips of *Q. michauxii*	CGMCC 42277
*Clitopilus cf. baronii*	XST	Roots of *Cymbidium* Golden (an orchid plant)	CGMCC 41176
*C. prunulus*	84496	Fruiting body	CFCC 84496
*Clitopilus cf. scyphoides*	50334	Fruiting body	CFCC 50334
*Clitopilus* cf. *prunulus*	HSL	ECM root tips of *Q. stewardii*	CGMCC 41599

Abbreviation: HKAS-Kunming Institute of Botany Academia Sinica; CFCC-Chinese Forestry. Culture Collection Center; CGMCC-China General Microbiological Culture Collection Center. ECM: ectomycorrhizal.

### Molecular phylogenetic analyses

Genomic DNA was extracted from 8-day-old free-living mycelia obtained on PDA overlaid with cellophane membrane. Total genomic DNA was extracted using a modified cetyltrimethylammonium bromide (CTAB) method (Li et al. 2024). The concentration and purity of the genomic DNA were determined using a Qubit fluorometer and Nanodrop 2000 spectrophotometer (Thermo Fisher Scientific, Carlsbad, USA). The DNA solution was stored at -20 °C for further molecular marker and genome sequencing. The nuclear DNA internal transcribed spacer region (ITS) is a commonly used genetic marker for inferring phylogeny (White et al. 1990). Closely related *Clitopilus* species sequences were obtained from a BLASTn search. All sequences were retrieved from the National Center for Biotechnology Information (NCBI) or genomic data. Sequence alignment was generated using MUSCLE v3.8.31 and then manually edited with MEGA v11 (Tamura et al. 2021). Under the Bayesian Information Criterion (BIC), Kimura 2-parameter was applied to find the best-fit substitution model, and the ITS sequence dataset comprised 607 characters, including gaps. Construction of the phylogeny was conducted using neighbor-joining (NJ) in MEGA v11 with 1,000 times self-expansion analyses. The phylogenetic tree file obtained was imported into the online visualization software Ghiplot (https://www.chiplot.online/) for annotation and editing.

### Genome sequencing, assembly, and annotations

Sequencing of *Clitopilus* genomic DNA was performed commercially by Novogene (Beijing, China). We sequenced strains HSL and NL-19 using a combination of PacBio Sequel II PacBio and the Illumina platform. Strain CAB genome was assembled using Illumina and Oxford Nanopore sequencing data. To improve the quality of the assembly, strains HSL, 84496, and XST genomes were assembled directly from PacBio HiFi long reads. Genome assembly quality was evaluated using Basidiomycota_odb10 lineage data containing 1,764 BUSCO (Benchmarking Universal Single-Copy Orthologs) groups. Genome assemblies and annotations of strains QYL-10 and DSM1602 have been previously reported (Peng et al. 2021; Schafhauser et al. 2022). However, the latter genome was re-annotated based on existing genomic information. Furthermore, transposable elements (TEs), protein-coding genes, secondary metabolism biosynthetic gene clusters (BGCs), and carbohydrate-active enzyme (CAZyme) repertoire were identified and annotated as described by Peng et al. (2021). TEs were identified using RepeatMasker v4.07 and Repbase database v23.06, and modelled *ab initio* using RepeatModeler v1.0.11 and LTR FINDER with default parameters. For accurate annotation of protein-coding genes, an approach combining homology-based, de novo *ab initio* prediction was used and integrated into a non-redundant and more complete gene set by the MAKER v2.31.9 annotation pipeline. Finally, genes were annotated using SwissProt, TrEMBL, KEGG, InterPro, and GO. Secondary metabolite BGCs were predicted using antiSMASH v4.0.2. To identify the candidate pleuromutilin BGCs, a BLAST search using terpene synthase from *Clitopilus pseudo-pinsitus* (accession number LC314149) as the query (Yamane et al. 2017) was employed. The syntenic relationships among the identified pleuromutilin BGCs were analyzed across eight *Clitopilus* strains. CAZymes were annotated using the dbCAN2 v2.0.11 web-based meta server (http://cys.bios.niu.edu/dbCAN2 ). HMMER v3.3 searches were performed against the dbCAN hidden Markov model (HMM) database, DIAMOND v0.9.24.125 searches were performed against the CAZy pre-annotated CAZyme sequence database (Buchfink et al. 2015), and Hotpep searches were performed against the conserved CAZyme short peptide database.

### Phylogenomics and comparative genomic analysis of CAZymes

All BUSCO-identified genes were clustered using OrthoFinder v2.3.8 to find single-copy gene families (Emms and Kelly 2019). After filtering short, low-quality genes (encoding proteins with < 200 amino acids), 957 single-copy genes were used to construct a phylogenetic tree. The final CDS alignment files of the single-copy gene family were concatenated into a supergene using MUSCLE v3.8.31 (Edgar 2004). RAxML v8.2.12 was used to reconstruct the maximum-likelihood (ML) tree using the optimal GTRGAMMAI substitution model and 1,000 bootstrap replicates (Stamatakis 2006). For the estimation of divergence times at each node in the phylogenetic tree, the Bayesian Markov-chain Monte Carlo (MCMC) tree module implemented in PAML v4.9 was employed, with the GTR nucleotide substitution model specified (Yang 2007). However, it should be noted that we did not attempt to calibrate the molecular clock because of the very limited fossil record of *Agaricales*. Principal component analysis (PCA) based on CAZyme gene counts was conducted with genomes from all *Clitopilus* strains, along with an additional 17 genomes from six brown-rot fungi taxa, five ECM/endophytic taxa, and six white-rot/other saprotroph taxa. All annotations were downloaded from the JGI MycoCosm database (Looney et al. 2022).

### DAPI staining of nuclei in hyphae

Hyphal nuclei were stained with 50 μL of 50 μg·mL^-1^ DAPI solution (4', 6-diamidino-2-phenylindole) for 20 min in the dark. The cell walls were then stained with 50 μL of 2.5 ng·mL^-1^ CFW solution (calcofluor white) for 5 min in the dark and washed three times with phosphate buffer saline (PBS, 0.1 M, pH 6.8) (Wei et al. 2024). Finally, the slides were observed using a confocal laser scanning microscope (Carl Zeiss, Jena, Germany) equipped with the ZEN2 software. The number of nuclei in each hyphal cell was determined.

### Growth rate and nutrient utilization pattern of *Clitopilus*

To measure the growth rate of *Clitopilus*, PDA plates inoculated with agar plugs were used to measure colony diameter using the crossline method (Li et al. 2024). To evaluate nutrient utilization patterns (carbon and nitrogen), a basal liquid medium was prepared as follows. The carbon-free basal medium contained (L^-1^): 1 g KH_2_PO_4_, 0.2 g MgSO_4_·7H_2_O, 0.02 g FeCl_3_·6H_2_O, 200 μg thiamine-HCl, 1 g yeast extract, 10 mL L^-1^ of the trace element stock solution containing 0.83 mg KI, 0.25 mg Na_2_MoO_4_·2H_2_O, 6.2 mg H_3_BO_4_, 0.025 mg CuSO_4_·5H_2_O, 16.9 mg MnSO_4_·H_2_O, 0.025 mg CoCl_2_·6H_2_O, 8.6 mg ZnSO_4_·7H_2_O. Twelve carbon sources, including monosaccharides (glucose, fructose, mannose, and mannitol), disaccharides (lactose monohydrate, trehalose, maltose, and sucrose), and polysaccharides (xylose, pectin, soluble starch, and xylan) were added to the carbon-free basal medium at a final carbon concentration of 3.375 g·L^-1^ (Qin et al. 2017). The nitrogen-free basal medium contained (L^-1^): 7.95 g glucose, 0.15 g MgSO_4_·7H_2_O, 0.05 g CaCl_2_, 0.25 g KH_2_PO_4_, 133 μg thiamine-HCl, 0.01 g Fe (III)-citrate, and trace elements (10 mL). The final nitrogen concentration was adjusted to 106 mg·L^-1^ using 12 nitrogen sources: (NH_4_)_2_SO_4_, KNO_3_, CO(NH_2_)_2_, valine (Val), leucine (Leu), phenylalanine (Phe), glutamine (Glu), glycine (Gly), bovine serum albumin (BSA, mean of 16% N), yeast extract (mean of 11.3% N), and peptone (mean of 14.5% N). Each flask was inoculated with three agar plugs (5.0 mm in diameter) and incubated in the dark at 25 °C for 5 weeks. Mycelial suspensions were collected, and the dried mycelial biomass was measured. Each assay was performed in four independent biological replicates.

### *In vitro* production of IAA and pleuromutilin

The culture conditions for fungal IAA production were determined as described by Kumla et al. (2020), and IAA measurements were conducted by a colorimetric assay (Tsavkelova et al. 2007). To quantify the pleuromutilin production, fungi were grown in a medium containing (L^-1^): 4 mL soybean oil, 50 g glucose, 12.5 g yeast extract, 1 g KH_2_PO_4_, 0.5 g MgSO_4_·7H_2_O, 0.7 g Ca(NO_3_)_2_·4H_2_O, 0.1 g NaCl, 0.05 g FeSO_4_·7H_2_O, and pH 6.3 at 25 °C with a shaking speed of 200 rpm (Hartley et al. 2009). Methanol was added to the freeze-dried mycelial mats and culture broths for 1 h to extract pleuromutilin, which was then decanted and filtered through Whatman No. 1 filter paper. Methanol was evaporated in vacuo in a rotary evaporator to obtain a crude extract of pleuromutilin (Hartley et al. 2009; Sun et al. 2017). Pleuromutilin content from mycelial fractions and fermentation broth fractions were determined using a UPLC-MS/MS system with an ACQUITY I-Class UPLC and an XEVO TQ-S micro triple quadrupole mass spectrometer (Waters Corp., Milford, MA, USA). The ACQUITY UPLC HSS T3 column (1.8 μm, 100 mm × 2.1 mm) was used with acetonitrile-water mobile phases at a flow rate of 0.3 mL·min^-1^ and a column temperature of 40 °C. The retention time of pleuromutilin was approximately 2.7 min. Pleuromutilin obtained from Shanghai Yuanye Bio-Technology Co., Ltd (Shanghai, China) was of analytical grade and used as the reference standard for analysis (1 μg·mL^-1^).

### Determination of five enzymes activities related to nutrient absorption

Seven strains were inoculated into liquid Pachlewski medium (L^-1^), which contained 0.5 g (NH_4_)_2_C_4_H_4_O_6_, 1.0 g KH_2_PO_4_, 0.5 g MgSO_4_, 20.0 g glucose, 0.1 mg vitamin B1, and 1 mL of the trace element stock solution (Yuan et al. 2004). Three fungal agar blocks were added to 100 mL of the medium and incubated at 25 °C with a shaking speed of 150 rpm. After one week, 100 mg of fresh filtered mycelia were homogenized in 1 mL of extraction buffer and centrifuged at 4 °C (15 min, 10,000 × g) and the supernatants were used to determine enzyme production using the commercial kits including Urease Assay Kit (ST5200, Angle gene), Acid Protease Assay Kit (ZM4280, Angle gene), Nitrate Reductase Assay Kit (SD2200, Angle gene), Chitinase Assay Kit (ANG-1136, Angle gene) and Acid Phosphatase Assay Kit (BC2135, Solarbio). These enzymes are crucial not only for symbiotic functioning, but also for the exploitation of mineral nutrients from soil organic matter (Pritsch and Garbaye 2011).

### Statistical analysis

All statistical analyses were conducted using analysis of variance (ANOVA) with post-hoc Tukey tests (*P* < 0.05). The data presented in this work were obtained using Origin 6.0 (enzyme activities and IAA production), ggplot2 in R Studio v4.0.3 (the fungal growth rate and nutrient utilization patterns), and GraphPad prism v9.5 (the bar plots show the total number of BGCs and CAZymes).

## Results

### Phylogenetic analysis and ecological metadata reveal potential lifestyle diversity in *Clitopilus*

**Figure 1.**
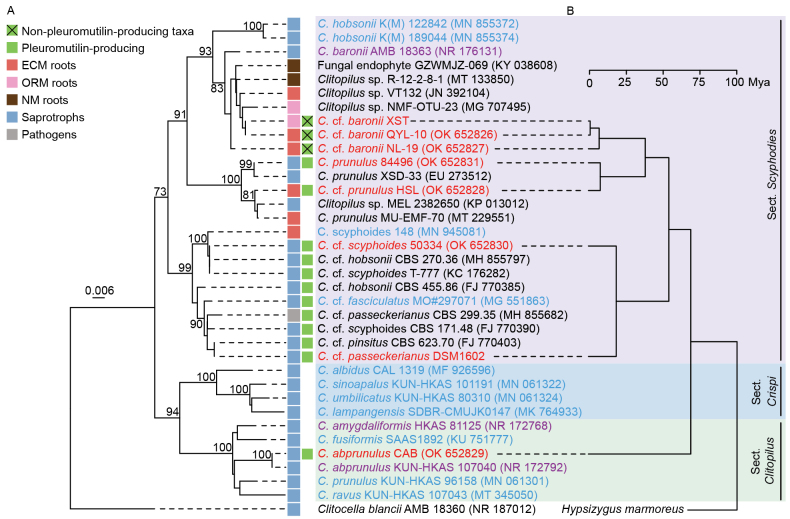
Phylogenetic relationships of *Clitopilus* species from the three sections. **A** The NJ tree based on ITS sequences. Bootstrap values of > 70% are shown for each branch. The potential lifestyles and pleuromutilin production of each taxon were mapped on the trees. **B** A phylogenomic analysis showing the evolutionary relationships among eight *Clitopilus* strains based on 957 single-copy orthologous genes. ECM: ectomycorrhizal; ORM: orchid mycorrhizal; NM: non-mycorrhizal. The taxa in purple are sequences from type strains. Taxa in blue are sequences from voucher sequences and taxa in red are sequenced in this study.

ITS-based phylogenetic analysis partitioned the *Clitopilus* taxa into three sections (Jian et al. 2020b, 2025), with our seven test strains assigned to sections *Clitopilus* and *Scyphoides* (Fig. 1A). To infer potential lifestyle modes, we compiled the habitat metadata for all taxa in the phylogeny. This topology was further corroborated by the phylogenomic analysis with divergence time estimations (Fig. 1B).

A striking ecological pattern emerged: multiple taxa within the section *Scyphoides* were recovered from living plant roots, including both mycorrhizal and non-mycorrhizal root systems, suggesting potential endophytic or mycorrhizal associations. Although direct experimental confirmation of symbiosis is currently lacking for most isolates, this pattern supports our previous finding that QYL-10 can establish biotrophic interactions with plants *in vitro* (Peng et al. 2021, 2022). In contrast, taxa from the other two sections were exclusively associated with soil and decaying wood, which is consistent with saprotrophic lifestyles. This phylogenetic clustering of habitat preferences suggests that transitions from saprotrophy to symbiotrophy lifestyles have occurred within *Clitopilus*, particularly in the section *Scyphoides*.

Host plant associations further support the potential symbiotic capacity. Several root-associated *Clitopilus* isolates were recovered from phylogenetically related hosts, including uncultured *Clitopilus* clone VT132, and strains NL-19, QYL-10, and HSL, colonizing *Quercus* roots, whereas uncultured *Clitopilus* clone NMF-OTU-23 and strain XST were detected in orchid roots. This host clustering suggests specialized plant-fungal associations rather than random root colonization. Previous studies have identified one clade within the section *Scyphoides* as pleuromutilin-producing species (Jian et al. 2020b). However, our expanded sampling revealed that pleuromutilin biosynthetic capacity is not restricted to saprotrophic lineages, but may also occur in taxa with endophytic or biotrophic tendencies (see genomic analyses below).

### High-quality genome assemblies enable comparative analysis of biosynthetic and degradative potential

Our sequencing strategy yielded high-quality haploid nuclear genome assemblies for all seven strains (Table 2). While the genomes of strains QYL-10 and DSM1602 have been previously published (Peng et al. 2021; Schafhauser et al. 2022), we re-annotated DSM1602 following gene de-redundancy to improve BUSCO completeness from the originally reported value to 98.6%. Assembled genome sizes ranged from 36 to 55 Mb, with variation primarily driven by transposable element (TE) content. Strain CAB showed exceptionally high TE proliferation (24.03% assembly). Notably, telomeric repeats were identified in assembled scaffolds, with strains XST and 50334 containing 10–11 telomere-to-telomere scaffolds with no sequencing gaps approaching chromosome-level contiguity. High BUSCO scores across all assemblies confirmed that genome quality was suitable for comparative analysis.

**Table 2.** Summary of genome assembly and annotation statistics of eight *Clitopilus* strains.

Assembly statistics	XST	QYL-10	NL-19	84496	HSL	DSM1602	50334	CAB
Total genome size (Mb)	37.31	36.93	39.77	53.85	54.53	65.62*	35.75	39.67
N50 contig length (Mb)	3.32	3.30	0.80	4.17	2.33	0.65	2.98	2.92
Contig numbers	13	13	112	20	33	167	14	47
Telomere sequences at both ends	11	10	2	6	2	0	10	6
Telomere sequences at one end	1	3	22	7	18	44	3	6
Total transposable elements (%)	4.6	3.9	5.9	13.5	11.2	10.2	4.9	24.0
Gene number	13,398	12,716	13,145	17,697	17,321	12,365	12,583	11,059
Complete BUSCOs (%)	94.6	98.4	96.8	95.7	98.2	98.6	97.1	93.4

*Note: the genome of DSM1602 derived from the dikaryon, probably leading to a larger genome size than those of the other haploids.

**Figure 2.**
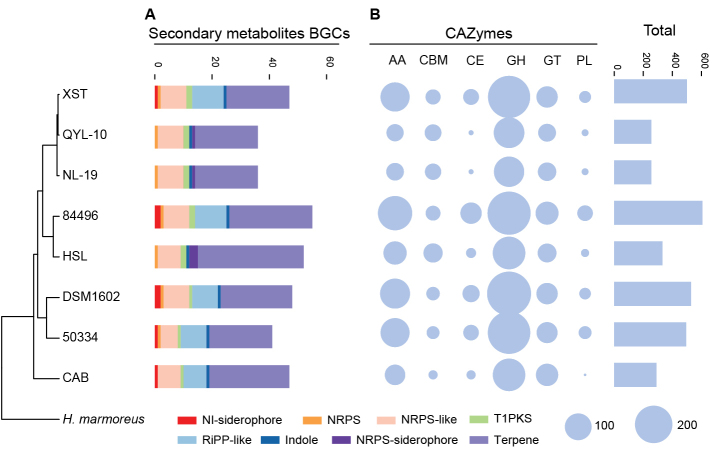
Characterization and comparative analysis of BGCs and CAZymes in *Clitopilus* spp. **A** Composition and number of BGCs in *Clitopilus* spp. Abbreviations: non-ribosomal peptide synthetase-independent siderophore (NI-siderophore), non-ribosomal peptide synthetases (NRPS), type 1 polyketide synthases (T1PKS), ribosomal post-translational peptides (RiPP-like). **B** Overview of CAZyme profiles of *Clitopilus* spp. The numbers of genes in different classes of CAZymes are shown and compared. Abbreviations: AA, auxiliary activitie; CBM, chitin-binding motif; CE, carbohydrate esterase; GH, glycoside hydrolase; GT, glycosyl transferase; PL, polysaccharide lyase.

Annotation of secondary metabolite BGCs revealed three major types commonly present across *Clitopilus* genomes: terpene, NRPS-like, and RiPP-like clusters, although strains HSL, NL-19, and QYL-10 lacked RiPP-like clusters (Fig. 2A). Terpene clusters were the most abundant, with strain HSL harboring 37 clusters, which was the highest number observed. Notably, BGC abundance varied substantially, even among closely related strains, suggesting a dynamic evolutionary turnover of secondary metabolic capabilities.

CAZyme repertoires, key determinants of fungal lifestyle and ecological niche (Looney et al. 2022; Martin and van der Heijden 2024), showed marked variation among strains (Fig. 2B). Strain 50334 possessed the highest total CAZyme encoding genes count, whereas strains QYL-10, NL-19, CAB, and HSL contained notably fewer CAZyme encoding genes, particularly AAs, CEs, and PLs. GHs and AAs are the most abundant classes of CAZymes across all genomes. Importantly, substantial intraspecific variation in both BGC and CAZyme counts were observed within the *Clitopilus cf. baronii* species complex, with strain XST containing higher numbers of both. This uncorrelated distribution of secondary metabolites BGCs and CAZymes with phylogenetic position suggests that genomic architecture and metabolic capabilities evolve independently of broad taxonomic groupings, potentially reflecting ecological adaptation.

### CAZyme repertoire analysis supports lifestyle diversity within *Clitopilus*

**Figure 3.**
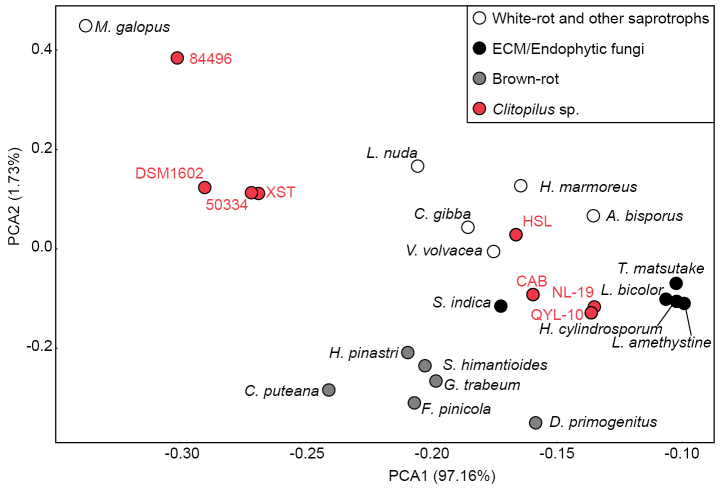
PCA plot of CAZyme profiles from 25 fungal genomes, including eight *Clitopilus* strains (indicated by solid red circles), six white-rot and other saprotrophs (indicated by open white circles), five ECM/endophytic fungi (indicated by solid black circles), and six brown-rot fungi (indicated by solid gray circles). *M. galopus*, *Mycena galopus* (Pers.) P. Kumm.; *L. nuda*, *Lepista nuda* (Bull.) Cooke; *H. marmoreus*, *Hypsizygus marmoreus* (Peck) H.E. Bigelow; *C. gibba*, *Clitocybe gibba* (Pers.) P. Kumm.; *A. bisporus*, *Agaricus bisporus* (J.E. Lange) Imbach; *V. volvacea*, *Volvariella volvacea* (Bull.) Singer; *T. matsutake*, *Tricholoma matsutake* (S. Ito & S. Imai) Singer; *L. bicolor*, *Laccaria bicolor* (Maire) P.D. Orton; *S. indica*, *Serendipita indica* (Sav. Verma, Aj. Varma, Rexer, G. Kost & P. Franken) M. Wei, Waller, A. Zuccaro & Selosse; *H. cylindrosporum*, *Hebeloma cylindrosporum* Romagn.; *L. amethystina*, *Laccaria amethystina* Cooke; *H. pinastri*, *Hydnomerulius pinastri* (Fr.) Jarosch & Besl; *S. himantioides*, *Serpula himantioides* (Fr.) P. Karst.; *G. trabeum*, *Gloeophyllum trabeum* (Pers.) Murrill; *C. puteana*, *Coniophora puteana* (Schumach.) P. Karst.; *F. pinicola*, *Fomitopsis pinicola* (Sw.) P. Karst.; *D. primogenitus*, *Dacryopinax primogenitus* D. J. McLaughlin & E. G. McLaughlin.

To contextualize *Clitopilus*CAZyme profiles within the broader landscape of fungal lifestyles, we conducted principal component analysis (PCA) using counts across six CAZyme categories (GH, AA, CE, PL, GT, and CBM) for 25 fungal genomes, including well-characterized white-rot, brown-rot saprotrophs, mutualistic ectomycorrhizal (ECM), and endophytic species (Kohler et al. 2015; Miyauchi et al. 2020). The first principal component (PC1) explained 97.16% of the total CAZyme variation, effectively capturing the major lifestyle-associated differences in the degradative enzyme repertoires (Fig. 3).

PCA revealed clear functional partitioning of *Clitopilus* strains into two distinct groups along PC1. Strains CAB, QYL-10, and NL-19 clustered with ECM and endophytic fungi, characterized by reduced plant cell wall-degrading enzymes typical of biotrophic lifestyles. Strain HSL occupied an intermediate position between the white-rot/soil saprotrophic fungi and the ECM/endophytic cluster, suggesting transitional or facultative capabilities. In contrast, strains 50334, XST, 84496, and DSM1602 were positioned closer to white-rot and other saprotrophic groups, indicating the retention of extensive lignocellulose-degrading capacity. Notably, all *Clitopilus* strains were distant from brown-rot fungi, consistent with the absence of Fenton chemistry-based wood decay mechanisms in this lineage.

Strikingly, strains within the *Clitopilus cf. baronii* species complex exhibited intraspecific lifestyle heterogeneity: strain XST clustered with saprotrophs, whereas closely related strains QYL-10 and NL-19 showed CAZyme profiles resembling biotrophic fungi. This intraspecific variation suggests that lifestyle transitions in *Clitopilus* may occur at fine evolutionary scales, potentially driven by ecological adaptation to different substrate or host conditions, rather than representing fixed species-level traits.

### Phenotypic and growth characteristics vary among *Clitopilus* strains

**Figure 4.**
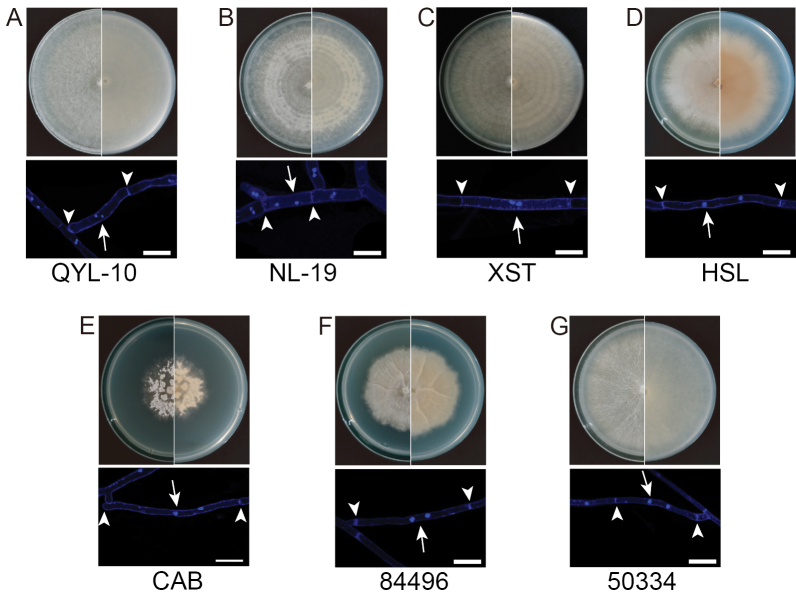
Diverse growth phenotypes of seven *Clitopilus* strains after 10 days at 25 °C on PDA plates. The colony appearance and two nuclei per hyphal compartment are shown. Arrows indicate nuclei and single arrowheads indicate septa. Scale bar: 10 μm.

**Figure 5.**
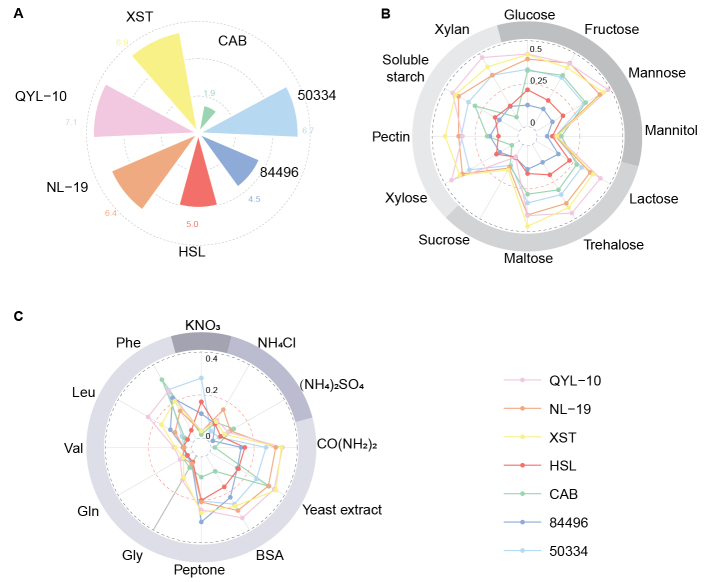
Growth behavior and nutrient utilization patterns of seven *Clitopilus* strains. **A** Colony diameters (cm) at 8 days post-inoculation on PDA plates. **B** Fungal biomass (mycelial dry weight, g/100 mL) when cultured on twelves carbon sources. **C** Fungal biomass (mycelial dry weight, g/100 mL) in response to twelves nitrogen sources. In the outer circle of B, carbon sources falling into monosaccharides, disaccharides, and polysaccharides are indicated by dimming shades of grey. Similarly, in the outer circle of C, nitrogen sources falling into ammonium, nitrate, and organic nitrogen are indicated by dimming shades of grey. All the values represent the average of four biological replicates.

Colony morphology on PDA revealed substantial phenotypic diversity among the seven *Clitopilus* strains (Fig. 4A–G). After 10 d of growth, strains QYL-10, NL-19, and XST displayed concentric zonation patterns. Strain HSL developed distinctive yellow-to-orange pigmentation in the aerial mycelium with dark brown coloration on the reverse side of the colony. Strain 84496 exhibited characteristic radial cracking, whereas strain CAB uniquely produced visible pustules or hyphal aggregates on the colony surface. All seven strains were dikaryotic but unexpectedly lacked clamp connections, consistent with previous morphological descriptions of *Clitopilus* (Dennis 1970; Noordeloos and Gates 2012; Asif 2025). Growth rate measurements revealed significant variations among strains (Fig. 5A). All *Clitopilus cf. baronii* strains (QYL-10, NL-19, and XST) exhibited faster radial expansion than the other tested species, while strain CAB showed the slowest growth. This growth rate hierarchy persisted across multiple media types, suggesting intrinsic metabolic differences, rather than medium-specific responses.

### Nutritional profiling reveals distinct carbon and nitrogen preferences

Fungal nutrient utilization patterns reflect ecological strategies adapted to local nutritional environments. Growth assays on defined media revealed shared preferences and strain-specific differences (Fig. 5B, C). Strains QYL-10, NL-19, and XST consistently produced higher biomass across all tested carbon and nitrogen sources than the other strains, which correlated with their faster growth rates. Carbon source utilization showed notable restrictions: all strains failed to grow on mannitol and sucrose, indicating that these are unsuitable carbon sources for *Clitopilus*. Strain CAB displayed particularly poor growth on xylose and xylan as the sole carbon sources (Fig. 5B). This suggests a reduced capacity for hemicellulose utilization, potentially reflecting ecological specialization. The nitrogen source preferences revealed a strong bias toward organic compounds. The yeast extract, BSA, and peptone supported robust growth across all strains, indicating efficient proteolytic activity. In contrast, nitrate utilization varied substantially: strains 50334 and HSL achieved maximal growth on nitrate as the sole nitrogen source, whereas other strains showed reduced nitrate assimilation capacity (Fig. 5C). This variation suggests divergent nitrogen acquisition strategies potentially related to soil versus plant-associated ecological niches, as plant symbionts often exhibit reduced reliance on inorganic nitrogen.

### Nutrient-acquiring enzyme activities show strain-specific profiles with elevated capacities in *Clitopilus cf. baronii*

**Figure 6.**
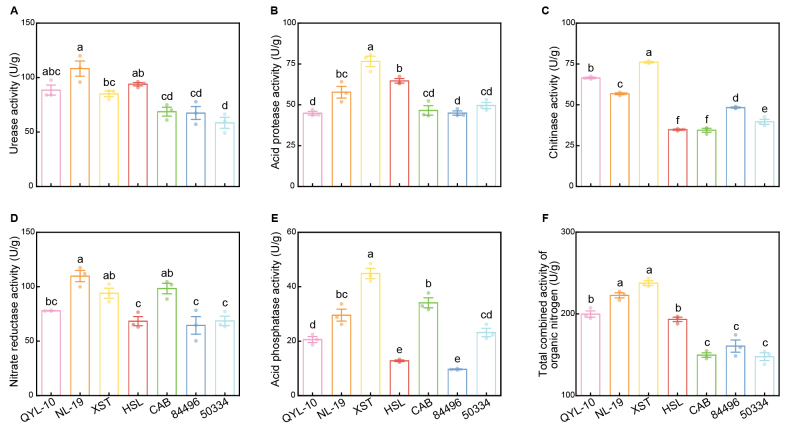
Five enzyme activities detected in seven *Clitopilus* strains, including nitrogen-metabolizing enzymes: urease (**A**), acid protease (**B**), chitinase (**C**), nitrate reductase (**D**), phosphorus-metabolizing enzymes: acid phosphatase (**E**), and total enzyme activities responsible for organic N mineralization (**F**).

Nitrogen and phosphorus availability frequently limits fungal and plant growth in soil ecosystems (Hu and Chu 2020). Fungi mobilize these nutrients through the enzymatic degradation of organic macromolecules, such as proteins, nucleic acids, and chitin, using urease, acid protease, chitinase, and acid phosphatase. Given the observed differences in nitrate utilization, we also measured nitrate reductase activity. Together, these five enzymes characterize nutrient acquisition strategies. Enzyme activity profiling revealed pronounced strain-specific patterns (Fig. 6A–E). Strain NL-19 exhibited the highest urease and nitrate reductase activities, significantly exceeding those of the other strains. Strain XST displayed significantly elevated acid protease, chitinase, and acid phosphatase activity (*P* < 0.05). Conversely, strains HSL and 84496 showed the lowest acid phosphatase activities. When extracellular enzymes involved in organic nitrogen mineralization (urease, protease, and chitinase) were considered collectively, a clear pattern emerged: strains within the *Clitopilus cf. baronii* complex, particularly NL-19 and XST, exhibited higher total nitrogen-mobilizing enzymatic capacity (Fig. 6F). However, phylogenetic mapping of enzyme activity patterns revealed no consistent correlation with evolutionary relationships, suggesting that enzymatic capabilities have evolved independently across lineages, potentially in response to ecological selective pressures, rather than phylogenetic constraints.

### IAA production occurs through a tryptophan-dependent biosynthetic pathway

**Figure 7.**
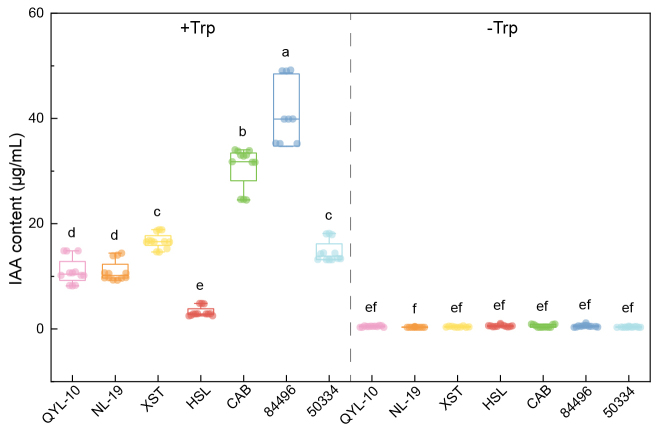
*In vitro*IAA production of seven *Clitopilus* strains in the presence (left panel) or absence of tryptophan (right panel), serving as a precursor for IAA biosynthesis. Trp: tryptophan. Based on these results, we further generated a table matching genomic and physiological traits (CAZyme loss and IAA production) to ecological predictions (e.g., saprotrophs vs. mutualists), as shown in Table 3.

IAA production by fungi can facilitate the establishment and maintenance of symbiotic associations with plants (Mehmood et al. 2019). To assess the potential for plant growth modulation, we quantified IAA production in all strains, with and without tryptophan supplementation (Fig. 7). Six of the seven strains (excluding strain HSL) produced detectable IAA exclusively in tryptophan-supplemented cultures, with no IAA detected in media lacking tryptophan (*P* < 0.05). This strict tryptophan dependency indicates that *Clitopilus* synthesizes IAA through the tryptophan-dependent indole-3-pyruvic acid pathway, which is the predominant route in plant-associated fungi (Sukumar et al. 2013; Mehmood et al. 2019). Strain 84496 exhibited the highest IAA production, approximately 2–4 fold greater than that of the other strains, followed by strain CAB. The inability of strain HSL to produce IAA even with tryptophan supplementation suggests the loss or downregulation of the biosynthetic pathway in this lineage, potentially reflecting reduced selective pressure for plant interaction capability. The widespread but variable capacity for IAA production across *Clitopilus* strains supports the hypothesis that plant-interactive capabilities are distributed throughout the genus, consistent with facultative biotrophic lifestyles.

### Pleuromutilin biosynthetic gene clusters show structural variation and wider distribution than previously recognized

**Figure 8.**
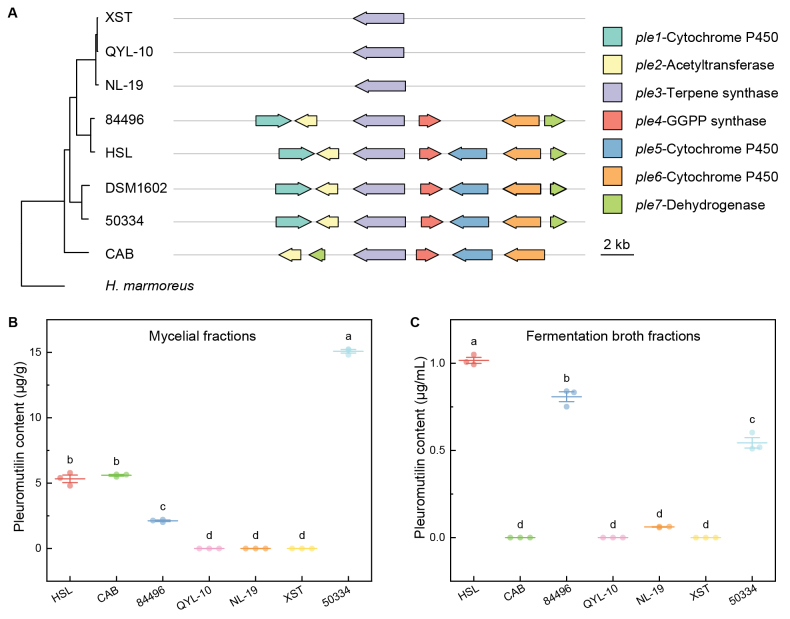
Pleuromutilin BGCs and pleuromutilin content of seven *Clitopilus* strains. **A** A typical pleuromutilin BGC, comprises seven genes, including *ple1*—cytochrome P450, *ple2*—acetyltransferase, *ple3*—terpene synthase, *ple4*—GGPP (geranylgeranyl pyrophosphate) synthase, *ple5*—cytochrome P450, *ple6*—cytochrome P450, and *ple7*—dehydrogenase. Syntenic analysis of the pleuromutilin BGC across seven *Clitopilus* strains. **B** Pleuromutilin content in the mycelial fractions. **C** Pleuromutilin content in fermentation broth fractions.

Pleuromutilin, a diterpenoid antibiotic with potent antibacterial activity, has been characterized from *C. passeckerianus* (Pilát) Singer strain DSM1602 and *C. pseudo-pinsitus* (Fr.) Joss. strain ATCC20527 (Yamane et al. 2017; Schafhauser et al. 2022). To assess the distribution and structural integrity of pleuromutilin BGCs across *Clitopilus*, we performed BLASTn searches against all seven genome assemblies. All seven strains contained recognizable pleuromutilin BGCs, although with substantial structural variations (Fig. 8A). Strains HSL and 50334 showed complete BGCs with high synteny with the DSM1602 reference. In contrast, the other strains displayed partial gene complements or structural rearrangements. The core terpene synthase gene *ple3* was conserved across all strains, indicating a strong selective retention of the initial cyclization step. However, tailoring enzyme genes showed variable presence: strain 84496 lacked *ple5* (encoding cytochrome P450), whereas strain CAB lacked *ple1* (encoding a different cytochrome P450) and exhibited inversion and translocation of *ple7* (encoding dehydrogenase). Most strikingly, all three *Clitopilus cf. baronii* strains (QYL-10, NL-19, and XST) retained only *ple3*, with apparent loss of all downstream tailoring enzymes. Chemical analysis of pleuromutilin production provided functional insights into the gene requirements (Fig. 8B, C). Strain 50334, HSL, and 84496 produced detectable pleuromutilin in both mycelial and extracellular (broth) fractions, with strain 50334 mycelia and strain HSL culture broth achieving the highest concentrations. Notably, strain 84496 produced pleuromutilin despite lacking *ple5*, indicating that this P450 enzyme is not essential for core pleuromutilin biosynthesis, although it may catalyze minor structural modifications. Strain CAB synthesized pleuromutilin intracellularly but showed no extracellular accumulation, suggesting that *ple1* or its associated genes may be involved in pleuromutilin transport or secretion. As predicted from their severely truncated BGCs, none of the three *Clitopilus cf. baronii* strains produced detectable pleuromutilin in either fraction. These findings expand the known distribution of pleuromutilin BGCs within *Clitopilus*.

## Discussion

This study provides the first comprehensive genomic and physiological characterization of the *Clitopilus* clade, revealing that ecological lifestyle diversity within this traditionally saprotrophic genus is underpinned by coordinated genomic and physiological adaptations. By integrating phylogenomics, CAZyme profiling, nutritional characterization, enzymatic assays, and secondary metabolite analysis across seven strains representing five species, we demonstrated that multiple *Clitopilus* lineages exhibit molecular signatures consistent with transitions from saprotrophy to plant-associated lifestyles (Martin and Tan 2025). These findings illuminate the mechanisms underlying the evolution of fungal symbiosis and position *Clitopilus* as a valuable model for studying lifestyle transitions in *Agaricales*.

### Genomic and physiological signatures of ecological adaptability

The pronounced variation in growth rates, colony morphology, and biomass production among *Clitopilus* strains reflects divergent ecological strategies. Rapid mycelial expansion in strains 50334, QYL-10, NL-19, and XST indicates aggressive nutrient acquisition, a trait advantageous in competitive saprotrophic environments, but also characteristic of facultative symbionts capable of rapid colonization (Ah-Fong et al. 2019). Carbon utilization patterns provide genomic evidence for ecological specialization. The inability of *Clitopilus* strains to utilize sucrose correlates with the absence of invertase genes (β-fructofuranosidases, GH32 family) in all sequenced genomes. GH32 gene presence correlates strongly with ecological strategy; saprotrophic fungi such as *Trichoderma* Persoon ex Gray spp. and most ECM fungi (Miyauchi et al. 2020) also lack GH32, whereas this gene family is expanded in many plant pathogens (Bergès 1993; Parrent et al. 2009; Khan et al. 2024). This shared absence across saprotrophic and mycorrhizal taxa, but not in pathogenic taxa, suggests that sucrose utilization is not essential for mutualistic plant associations, where fungi access different carbon sources from the host plant through controlled metabolite exchange rather than aggressive cell wall degradation. Similarly, the inability to metabolize mannitol likely reflects the non-expression or pseudogenization of genes encoding mannitol 1-phosphate dehydrogenase despite their genomic presence. This suggests regulatory rather than genetic constraints on metabolic capacity, potentially reflecting adaptation to habitats where mannitol is not the primary carbon source. Substantial inter- and intraspecific variation in enzymatic activities for nutrient acquisition indicates habitat-specific optimization, with enzyme expression profiles potentially reflecting local substrate availability. Most strikingly, the apparent loss or severe degradation of pleuromutilin biosynthetic gene clusters in *Clitopilus cf. baronii* strains, which show genomic and physiological signatures of plant association, suggests that antibiotic production may be selectively neutral or even disadvantageous in plant-symbiotic niches (Kohler et al. 2015; Miyauchi et al. 2020). Antibiotics facilitate competitive interactions in soil microbial communities, but establishing mutualistic plant associations may require compatible chemical signaling and tolerance of plant-beneficial microbiomes (Bills et al. 2013). This trade-off between antibiotic production and symbiotic capacity warrants further investigation as a potential driver of lifestyle transition.

### Nitrogen metabolism: from substrate preferences to molecular mechanisms

Nitrogen availability frequently limits fungal growth in terrestrial ecosystems, and nitrogen acquisition strategies often differ between saprotrophic and symbiotic fungi (Martin and van der Heijden 2024). The strong preference of all *Clitopilus* strains for organic nitrogen sources reflects their evolutionary history as decomposers. However, substantial inter- and intraspecific variations in nitrogen-metabolizing enzyme activities suggest an ongoing ecological differentiation. Strains NL-19 and XST exhibited the highest organic nitrogen-mineralizing activities (combined urease, protease, and chitinase), correlating with their superior biomass production and potentially reflecting their adaptation to protein-rich substrates in their native habitats. Conversely, QYL-10 showed comparatively lower enzymatic activities despite similar growth rates, suggesting alternative nitrogen acquisition strategies or metabolic efficiency differences. These intraspecific metabolic and growth heterogeneities may be shaped by their different host plants and genomic heterozygosity. The complex relationship between nitrate utilization and molecular machinery reveals a multilayered regulation. Four strains (QYL-10, NL-19, XST, and CAB) showed poor growth on nitrate, despite high nitrate reductase activity. Previous work demonstrated that strain QYL-10 lacks genes encoding high-affinity nitrate transporters within the *fHANT-AC* gene cluster (fungal high-affinity nitrate assimilation cluster), which in ECM fungi like *Laccaria bicolor* (Maire) P.D. Orton coordinates nitrate uptake and assimilation (Kemppainen et al. 2010; Kemppainen and Pardo 2013; Peng et al. 2022). Genomic analysis confirmed that all seven *Clitopilus* strains lacked canonical nitrate transporter genes, yet strains HSL, 50334, and 84496 achieved robust growth on nitrate despite low nitrate reductase activity. This apparent paradox suggests compensation through alternative transport mechanisms, potentially involving ATP-binding cassette (ABC) transporters or other non-canonical nitrate uptake systems (Maeda and Omata 2009), although this hypothesis requires experimental validation.

These findings demonstrate that nitrate utilization efficiency cannot be predicted from enzyme activity or transporter gene presence alone but instead reflects the complex regulatory integration of uptake, assimilation, and metabolic flux. Importantly, nitrogen source availability governs the establishment and maintenance of symbiosis in *Clitopilus*-plant associations: strain QYL-10 forms mutualistic associations with poplar under organic but not inorganic nitrogen conditions (Peng et al. 2022). Understanding the molecular mechanisms of nitrogen sensing, transport regulation, and metabolic reprogramming during plant colonization is a key priority in elucidating lifestyle transition mechanisms (Zhang et al. 2024).

### Convergent evidence for plant-associative capacity: IAA production and CAZyme repertoire remodeling

Two independent lines of evidence support the hypothesis that multiple *Clitopilus* lineages possess plant-interactive capabilities; phytohormone production and carbohydrate-active enzyme repertoire restructuring (Table 3).

**Table 3.** Matching IAA production and CAZymes loss with lifestyle predictions.

Strains	IAA production	Number of CAZymes encoding genes	Predicted symbiotic potentials with plants
QYL-10	+	△	M
NL-19	+	△	M
XST	+	△△△	S
HSL	-	△△	S/M
CAB	++	△△	M
84496	+++	△△△	S/M
50334	+	△△△	S

Note: Average IAA production was shown by “+”. “-” indicated no detectable signal, “+” indicated 5–20 μg/mL, “++” indicated 20–35 μg/mL and “+++” indicated more than 35 μg/mL. Genes count encoding CAZymes were shown by “△”. “△” indicated 200–300, “△△” indicated 300–400 and “△△△” indicated more than 400. “M” indicated mutualistic interactions, “S” indicated saprotrophic interactions and “S/M” indicated facultative mutualistic and saprotrophic interactions with plants.

#### IAA production as a symbiotic signal

Six of the seven strains produced IAA in a strictly tryptophan-dependent manner, indicating the utilization of the indole-3-pyruvic acid biosynthetic pathway predominantly in plant-associated fungi (Sukumar et al. 2013; Mehmood et al. 2019). Fungal IAA functions as a crucial signaling molecule initiating morphological and physiological changes in plant roots that facilitate symbiosis establishment, including lateral root proliferation and Hartig net formation during ECM colonization (Sun et al. 2014). Strains 84496 and CAB exhibited particularly high IAA production, suggesting strong potential for root architecture modulation and mutualistic interaction establishment (Spaepen et al. 2007; Fu et al. 2015). The failure of strain HSL to produce IAA even with tryptophan supplementation indicates the evolutionary loss of this pathway, potentially reflecting reduced selection for plant interaction in its ecological niche or functional redundancy with other signaling mechanisms.

#### CAZyme repertoire contraction as a lifestyle indicator

PCA of CAZyme profiles revealed functional partitioning of *Clitopilus* strains along the saprotrophy–biotrophy continuum. Strains CAB, QYL-10, and NL-19 clustered with ECM and endophytic fungi, characterized by contracted repertoires of plant cell wall-degrading enzymes, particularly reduced auxiliary activities (AAs), carbohydrate esterases (CEs), and polysaccharide lyases (PLs). This pattern mirrors transitions from saprotrophy to symbiotrophy documented across diverse fungal lineages (Hess et al. 2018; Martino et al. 2018; Martin and Tan 2025). CAZyme reduction in symbiotic fungi reflects evolutionary constraints; aggressive plant cell wall degradation is incompatible with mutualism, as it would harm the host. Instead, symbionts retain enzymes for storage, carbohydrate metabolism, and fungal cell wall remodeling while losing their lignocellulose-degrading capacity. The ECM-like structures formed by strain QYL-10 during poplar colonization *in vitro* (Peng et al. 2022) provided functional validation of the symbiotic potential predicted by its CAZyme profile. Conversely, strains XST and 50334 clustered with white-rot saprotrophs, indicating the retention of extensive lignocellulose-degrading capacity (Kellner et al. 2014; Miyauchi et al. 2020). Strain 84496 showed affinity for *Mycena galopus* (Pers.) P. Kumm., a facultative biotroph representing a transitional state between saprotrophy and root symbiosis (Harder et al. 2023), consistent with its intermediate position in our analyses. Intriguingly, strain CAB is phylogenetically positioned outside the canonical pleuromutilin-producing clade (Jian et al. 2020b), but synthesizes pleuromutilin while showing CAZyme profiles consistent with plant association. This mosaic of traits (antibiotic production capacity + symbiosis-compatible CAZome) exemplifies the transitional nature of lifestyle evolution, in which genomic features associated with different ecological strategies coexist during evolutionary shifts.

The extensive CAZyme repertoires retained by some *Clitopilus* strains resemble those of orchid and ericoid mycorrhizal fungi, which maintain broader enzymatic capabilities than ECM fungi and can switch between saprotrophic and symbiotic modes depending on the environmental conditions (Miyauchi et al. 2020; Gong et al. 2023). This functional flexibility may enable *Clitopilus* species to occupy diverse ecological niches within forest ecosystems, functioning as decomposers when plant partners are unavailable, but capable of forming facultative associations when conditions favor symbiosis.

### Evolutionary implications and future directions

Our findings reveal that *Clitopilus* encompasses a spectrum of ecological lifestyles, with multiple lineages, particularly within the section *Scyphoides*, exhibiting coordinated genomic and physiological adaptations consistent with transitions toward plant-associated lifestyles. The correlation between CAZyme profile remodeling, IAA production capacity, and habitat associations (recovery from living plant roots) provides convergent evidence that these transitions are ongoing or recently completed in evolutionary time.

Several key questions have emerged from this study. First, what selective pressures drive lifestyle transitions in *Clitopilus*? The loss of pleuromutilin biosynthetic capacity in putative plant-associated lineages suggests potential trade-offs between antibiotic production (advantageous for soil competition) and symbiotic compatibility (requiring chemical signaling conducive to mutualism). Second, do the observed genomic and physiological differences translate into functional symbioses in nature? While strain QYL-10 forms associations with poplar *in vitro*, field validation of symbiotic capacity across diverse *Clitopilus* strains and potential plant hosts is essential. Third, how do environmental conditions, particularly nitrogen availability, regulate the switch between saprotrophic and symbiotic modes? The nitrogen dependence of QYL-10’s symbiotic behavior suggests environmental control of lifestyle expression.

Addressing these questions requires integrative approaches; co-inoculation experiments with diverse plant hosts across soil conditions, transcriptomic profiling during colonization to identify symbiosis-activated gene networks, comparative genomics expanded to additional *Clitopilus* species to map trait evolution, and field surveys to document natural associations and ecological contexts. Particular attention should be paid to the apparent intraspecific variation in lifestyle within the *C. cf. baronii* complex, where closely related strains exhibit divergent CAZyme profiles and metabolic capabilities, suggesting that lifestyle is not a fixed species-level trait but an evolvable ecological strategy capable of responding to local selection pressures.

## Conclusions

This comprehensive characterization of *Clitopilus* genomes, physiology, and secondary metabolism provides several key insights. First, *Clitopilus* exhibits greater ecological diversity than previously recognized, encompassing both saprotrophic and facultative biotrophic lineages. Second, lifestyle diversity is underpinned by coordinated genomic (CAZyme repertoire remodeling, pleuromutilin BGC loss) and physiological (nitrogen preferences, IAA production) adaptations. Third, lifestyle transitions appear to occur at fine evolutionary scales, with intraspecific variation suggesting ongoing adaptation rather than ancient fixed divergences. Fourth, *Clitopilus* represents a valuable model system for studying the early stages of symbiosis evolution, capturing lineages at various points along the saprotrophy–biotrophy continuum.

Most importantly, by revealing the genomic and physiological toolkit underlying lifestyle transitions, this study illuminates the mechanisms through which fungi evolve from free-living decomposers into intimate plant partners, a fundamental evolutionary transition shaping terrestrial ecosystem functions.

## Additional information

### Use of AI

No use of AI was reported.

### Adherence to national and international regulations

All the fungal strains used in this study have been legally obtained, respecting the Convention on Biological Diversity (Rio Convention).

### Author ORCIDs

Yuwei Zhang https://orcid.org/0000-0002-9328-6428

Yuchen Wang https://orcid.org/0000-0003-1045-712X

Irina S. Druzhinina https://orcid.org/0000-0003-2821-5268

Zhilin Yuan https://orcid.org/0000-0002-5799-8401

Francis M. Martin https://orcid.org/0000-0002-4737-3715

### Data availability

Raw genomic data and assembled sequences were submitted to NCBI with BioProject accession numbers PRJNA1303054 and BioSample accession numbers SRR34903549, SRR34918758, SRR34922171, SRR34922172, SRR34931726, SRR34931745, and SRR34947942. Accession numbers for the assembled genomes are JBQGVY000000000, JBQGVZ000000000, JBQGWB000000000, JBQKMH000000000, JBQGWA000000000, JBQSUV000000000. All other data are available in the main text.
